# ^11^B NMR of the Morphological Evolution of Traditional Chinese Medicine Borax

**DOI:** 10.3390/molecules29010251

**Published:** 2024-01-03

**Authors:** Qiulin Li, Yawen Yang, Qingfeng Wang, Xiang Han, Junfeng Zhu, Nan Zhang, Qiuhong Wang, Kanshe Li, Pin Gong, Fuxin Chen

**Affiliations:** 1 Department of Chemistry and Chemical Engineering, Xi’an University of Science & Technology, Xi’an 710054, China; li_qiulin@163.com (Q.L.);; 2 School of Food and Biological Engineering, Shaanxi University of Science & Technology, Xi’an 710021, China; 3 Department of Safety Science and Engineering, Xi’an University of Science & Technology, Xi’an 710054, China

**Keywords:** boron-containing TCM, ^11^B NMR, borax, boric acid, boron-containing compound

## Abstract

This article applies nuclear magnetic resonance technology to the study of boron-containing traditional Chinese medicine, in order to explore the morphological evolution of boron elements in traditional Chinese medicine. Borax is a traditional Chinese medicine with anti-corrosion, anti-inflammatory, antibacterial, and anticonvulsant effects. It is made by boiling, removing stones, and drying borax minerals like borate salts. This article introduces an ^11^B nuclear magnetic resonance method for identifying and characterizing boron-containing compounds in TCM. We applied this technology to borax aqueous solutions in different chemical environments and found that with boron mixed in the form of SP^2^ hybridization in equilateral triangles and SP^3^ hybridization in equilateral tetrahedra, the pH changes in alkaline environments significantly affected the ratio of the two. At the same time, it was found that in addition to the raw material peak, boron signals of other boron-containing compounds were also detected in 20 commercially available boron-containing TCM preparations. These new boron-containing compounds may be true pharmaceutical active ingredients, and adding them directly to the formula can improve quality and safety. This article describes the detection of ^11^B NMR in boron-containing traditional Chinese medicine preparations. It is simple, non-destructive, and can provide chemical fingerprint studies for boron-containing traditional Chinese medicine.

## 1. Introduction

The culture of Chinese medicine has endured throughout China’s long history and is widely used in clinical applications even now. The Yellow Emperor’s Classic of Internal Medicine, Zhang Zhongjing’s Treatise on Typhoid, and Li Shizhen’s Compendium of Materia Medica all highlight the charm of Chinese medicine. The progress of modern technology has improved the level of preparation of traditional Chinese medicine and given it a more vigorous vitality. Borax has the effects of cooling and detoxifying, removing accumulated blocks, removing phlegm and heat, and reducing inflammation and ulcers [1]. Its pharmacological effects include anti-tumor effects [2], antifungal and antiviral effects [3], disinfection and anti-corrosion effects [4], and effects on body metabolism [5]. There are many experimental cases indicating that it is also an important antidote to animal fluorosis, which can reduce and delay bone fluoride accumulation, and correct excessive calcium phosphorus imbalance [5,6]. It can be seen that borax plays an important role in TCM treatment, and the study of borax in TCM has a certain reference value for the development of traditional Chinese medicine theory and the combination of traditional Chinese and Western medicine treatment. This paper mainly focuses on a large number of a class of TCM for boron-containing TCM preparations. In the TCM prescription database, there is 1 prescription involving boric acid and 365 prescriptions of borax. In the database of proprietary Chinese medicine prescriptions contains 19 prescriptions involving boric acid and 137 prescriptions involving borax. These preparations usually use borax or boric acid or even both as one of the rulers and adjuvants and are concocted into a series of Chinese medicinal preparations with medicinal value. This paper investigates whether the main boron-containing active ingredients in 20 commercially available boron-containing Chinese patent medicines have changed.

Nuclear magnetic resonance (NMR) spectroscopy has evolved into a powerful tool in drug discovery over the last two decades. Two-dimensional COSY ^11^B NMR spectroscopy can be used to identify the isomerism of boron-containing cluster compounds [7]. Park et al. found that isotropic ^11^B NMR chemical shifts from the borosilicate minerals correlated with the local geometric parameters of the tetrahedrally coordinated B sites [8]. Ksenofontov et al. provide a convenient tool and database that we collected for all researchers, that allows us to predict the ^11^B NMR chemical shift of boron-containing dyes [9]. Reto Horst et al. describe NMR spectroscopy as the Swiss army knife of drug discovery [10]. NMR can also have some more interesting applications in the field of traditional Chinese medicine. For example, Wen-Neng Lin et al. used ^1^H-NMR spectroscopy for chemical fingerprinting of dried ginseng root samples with cultivation ages ranging from 1 to 6 years. A distributed model was developed, which can be accurately applied to the assessment of the true cultivation age of various dried white ginseng root samples and commercial products [11]. The non-destructive nature of NMR offers the potential to observe protein–ligand interactions in a more complex environment. Some examples from the early stages of target preparation and hit discovery are summarized by Sébastien Keiffer et al. [12]. Therefore, NMR as a means to study the elemental morphological changes of boron in boron-containing Chinese medicinal preparations is undoubtedly a very feasible research method. Currently, there are two main types of boron-containing ingredients in boron-containing TCM preparations on the market: borax and boric acid, of which borax is the main ingredient, but it is not known whether the main active ingredient in the human body is still borax or whether other elemental forms of boron exist. At present, the experimental parameters and chemical shift data of ^11^B NMR are being improved, and the conversion between different elemental forms of boron can be well discovered by a chemical shift, which is the main research tool of this work.

Boron has an exceptional position in the periodic table; it is a second period III main group element, and the boron central atom can be transformed from sp^2^ to sp^3^ hybridization, as in the case of the tetrahedral borate anion B(OH)_4_^−^ [13]. The boron atom has an empty orbital and boron can form coordination covalent bonds with biological nucleophiles (e.g., hydroxyl and amine groups in enzymes, carbohydrates, and nucleic acids). Two isotopes of boron nuclei can produce NMR signals [14], ^10^B and ^11^B; 10B has a natural abundance of 19.9% and a sensitivity of 1.99 × 10^−2^ concerning ^1^H; ^11^B has a natural abundance of 80.1% and a sensitivity of 0.17 concerning ^1^H; both nuclei have a spin quantum number I greater than 1/2 and have quadrupole moments, so the NMR boron spectra (B NMR) spectral lines are wider. Chromatographic methods for boron analytical detection have been reported in the literature: ICP-MS detection of boron less than 1 ppm requires the instrumentation to be adapted for DMSO [15]. Also, LC in combination with MS can be used. Based on LC/MS/MS, Baldwin, et al. [16] developed a derivatization technique, and this method allows the trace detection of nitrogen-free aromatic boronic acid boron compounds with detection limits as low as 1–5 ppm. However, the selection of derivatization reagents and derivatization reactions is often time consuming, and sometimes pure standards are difficult to obtain. The ^11^B NMR technique has been applied to the synthesis and characterization of boron-containing compounds [17,18,19], as it does not require derivatization and is efficient and nondestructive, so we attempted to use the NMR technique for the analytical determination of boron-containing compounds.

Studies have shown that boron-containing compounds have antibacterial, anti-inflammatory, antioxidant, and anti-cancer characteristics, and affect the cellular matrix [13,20,21,22]. The traditional claim that “boron-based compounds are toxic and unstable” has been debunked [23], and boron-containing drugs have great promise through the substitution of specific pharmacological groups or chemical modifications [24,25,26,27]. Tuğba Erkmen evaluated the anti-leukemic effects of two promising boron compounds, borax pentahydrate (BP) and disodium pentaborate decahydrate (DPD), and compared their ability to trigger apoptosis in acute promyelocytic leukemia cells (HL-60) [28]. In 2003, bortezomib was approved by the FDA as the first organoboron drug for the treatment of multiple myeloma [29], and tavaborole [30] was used as a topical antifungal drug. Drug safety was studied according to ICHM7 guidelines and in 2011, O’Donovan et al. [31] reported boric acid as a novel bacterial mutagen and that 12 of the 13 boric acids and boronic esters evaluated showed mutagenic signs in Ames tests. Recently, boron purposes have been pro-longed further, with boronic acids/esters utilized as the de-fending crew in anticancer prodrugs that set off in excessive ROS tumor environments, and as doable nanocarriers to transport pills throughout the cell membrane [32]. Related animal experiments showed [33] that subacute boric acid was able to degrade the kidneys of mice [34], and that boron exhibited some reproductive toxicity in rats, mice, and dogs, i.e., inhibiting sperm fertilization at low doses and reducing epididymal sperm count at high doses [34,35,36,37]. 

Based on the above research background, we have developed an interest in exploring the elemental morphology evolution of boron in the processing of traditional Chinese medicine, borax, and boric acid. To our knowledge, there have been no studies using ^11^B NMR for the study of boron-containing TCM. In this article, we aim to describe the chemical behavior of boron atoms in borax in different chemical environments through the study of ^11^B NMR, detect boron signals in commercially available TCMs, and observe for the presence of new boron-containing active small molecules other than raw materials such as borax or boric acid. These new boron-containing compounds may be the true active ingredients in boron-containing traditional Chinese medicine, providing inspiration and reference for the effective detection of boron in drugs.

## 2. Results

### 2.1. Factors Affecting Chemical Shift

#### 2.1.1. ^11^B NMR Study of Borax Solutions at Various Concentrations

In this paper, we used BF_3_ as an external standard for the correction of chemical shifts and performed ^11^B NMR on different concentrations of borax. The chemical shifts of ^11^B are known to vary with its chemical environments, such as spatial site resistance and the shielding effect of electrons outside the nucleus. In general, an increase in spatial site resistance leads to a shift of the chemical shift of boron to the lower field, while an increase in the electron-giving group leads to a shift in the chemical shift of boron to the higher field. 

**Figure 1:**
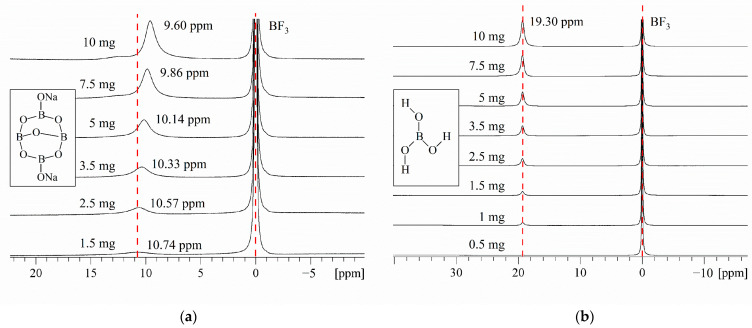
(**a**) ^11^B NMR shifts of dissolving 10 mg, 7.5 mg, 5 mg, 3.5 mg, 2.5 mg, and 1.5 mg of borax in 0.5 mL D_2_O; (**b**) ^11^B NMR shifts of dissolving 10 mg, 7.5 mg, 5 mg, 3.5 mg, 2.5 mg, 1.5 mg, 1 mg and 0.5 mg of boric acid in 0.5 mL D_2_O. The reference signal of BF_3_ at δ = 0.00 ppm is shown.

Figure 1 shows the ^11^B NMR shifts of dissolving 10 mg, 7.5 mg, 5 mg, 3.5 mg, 2.5 mg, and 1.5 mg of borax in 0.5 mL D_2_O, and the ^11^B NMR shifts of dissolving 10 mg, 7.5 mg, 5 mg, 3.5 mg, 2.5 mg, 1.5 mg, 1 mg, and 0.5 mg of boric acid in 0.5 mL D_2_O. We found an interesting phenomenon that the chemical shift belonging to borax shifts to the high field in the ^11^B NMR spectra with increasing borax concentration, decreasing from 10.74 ppm to 9.60 ppm, while in the ^11^B NMR spectra of D_2_O samples of anhydrous borax, the chemical shift of ^11^B NMR is 13.09 ppm. The difference between the ^11^B NMR spectra of different concentrations of borax and anhydrous borax is probably because of the different water content within the molecule and thus the different solventized molecules, i.e., the different chemical environment of ^11^B; we tentatively set the chemical shifts of borax in the ^11^B NMR spectra as a range, i.e., δ_B_ 9.60–13.09 ppm.

#### 2.1.2. ^11^B NMR Study of Borax Solutions at Various pHs

**Figure 2:**
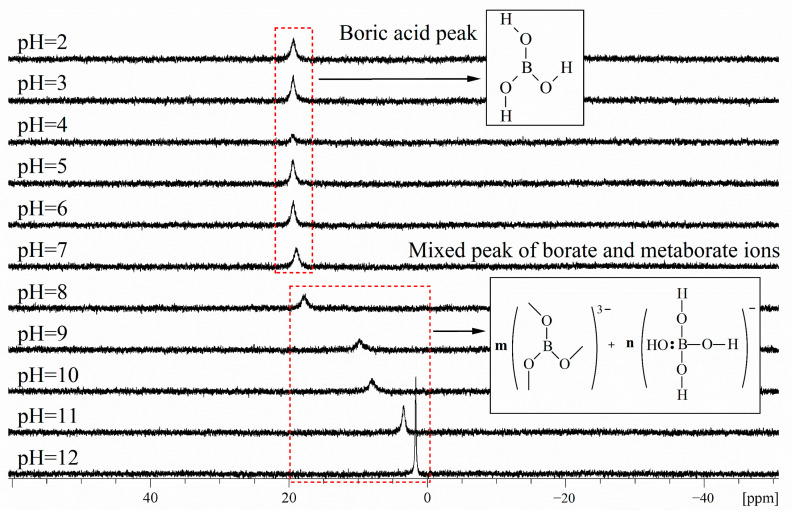
^11^B NMR spectra of borax solutions at pH 2, pH 3, pH 4, pH 5, pH 6, pH 7, pH 8, pH 9, pH 10, pH 11, and pH 12. The boron concentration of each solution was 0.052 M.

The pH of the borax aqueous solution is stable at around 9.2. We conducted ^11^B NMR on borax under different pH environments, and the results are shown in Figure 2. It was found that in acidic environments, the form of borax in aqueous solution remained stable as boric acid, and the ^11^B NMR detection result remained stable at around 19.30 ppm. When the alkaline substance NaOH is added to the borax aqueous solution, the ^11^B NMR detection results gradually shift towards a higher field with the increase in pH value. We speculate that the addition of NaOH leads to an increase in electron-donating groups, which causes the physical displacement of boron to shift towards a higher field.

Figure 2 shows the ^11^B NMR spectra of borax solutions at pH 2, pH 3, pH 4, pH 5, pH 6, pH 7, pH 8, pH 9, pH 10, pH 11, and pH 12. When pH > 7 and with the increase in OH- concentration, the concentration of borate ions increases and the concentration of metaborate ions decreases, In Figure 2, m gradually increases and n gradually decreases. Due to boron being an electron-deficient atom, in three coordinated boron compounds, boron atoms form three covalent bonds with atoms of other elements, leaving an empty p-orbital. Therefore, it can also accept a pair of electrons from other negative ions or molecules to form coordination bonds. When forming coordination bonds, boron atoms form bonds in sp3 hybrid orbitals with a tetrahedral spatial configuration. To prevent the possible influence of halogen atoms on the elemental form of boron during the pH adjustment process, use NaOH and acetic acid. The existence form of borax in alkaline environments can be distributed in the following three steps:

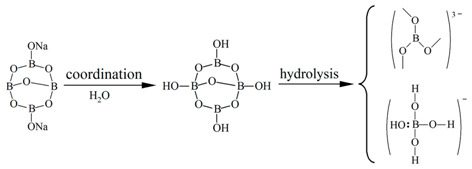

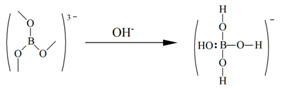

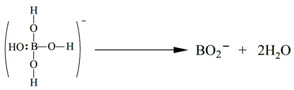



Borax undergoes a certain degree of coordination reaction in aqueous solution, and some SP^2^ hybridized boron atoms will transform into SP^3^ hybridized boron atoms due to the coordination reaction. In acidic environments (pH < 7), the elemental form of boron can be determined as the uncoordinated SP^2^ hybridized boron atom form, i.e., borate ions. In the process of gradually adjusting the solution environment to alkaline, more and more boron forms coordination bonds with a pair of electrons in OH^−^ due to the formation of empty orbitals, which are hybridized from SP^2^ to SP^3^, and the spatial configuration is transformed from a planar triangle to a spatial tetrahedron. As the number of SP^3^ hybridized boron atoms increases, the ^11^B NMR signal of the aqueous solution gradually shifts towards a higher field. When the pH was adjusted to 12, δ_B_ reached 1.63 ppm, at which point the boron atoms in the aqueous solution mainly existed in the form of SP^3^ hybridization. At this point, borate ions were transformed into metaborate ions, which is consistent with Park et al. [38], and corresponds to the ^11^B NMR signal of sodium metaborate measurement of δ_B_ = 1.3 ± 0.5 ppm.

Similar conclusions also appeared in the study by Islam et al. [39] in 2007. They researched the ^11^B NMR spectra of p-carboxy benzene boronic acid (PCBA) ions and demonstrated that their chemical shifts depend on the pH. They used NaOH and HCl for pH regulation, and they concluded that in an acidic solution, the boronic acid group of PCBA and the boronic anion exist as the same trigonal type. On the other hand, in an alkaline solution above pH 10, the group exists as a tetrahedral type.

#### 2.1.3. ^11^B NMR Study of Borax Solutions at Various Temperature

**Figure 3:**
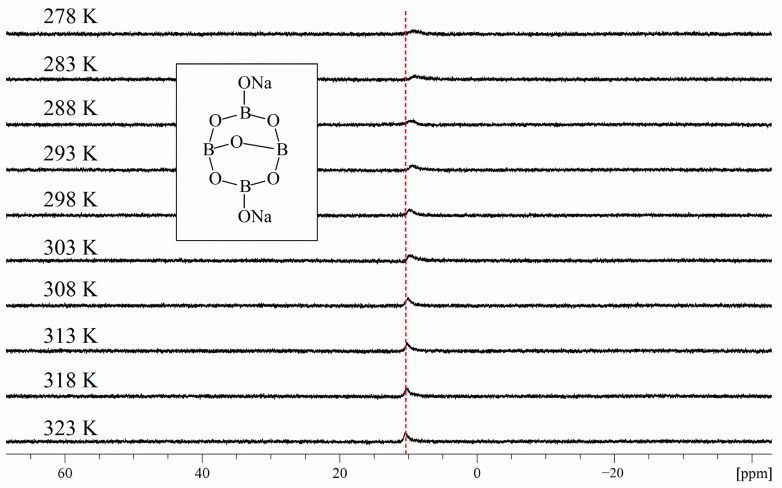
^11^B NMR spectra of borax solutions at 278 K, 283 K, 288 K, 293 K, 298 K, 303 K, 308 K, 313 K, 318 K, and 323 K. The boron concentration of each solution was 0.052 M.

Figure 3 shows ^11^B NMR spectra of borax solutions at 278 K, 283 K, 288 K, 293 K, 298 K, 303 K, 308 K, 313 K, 318 K, and 323 K. The boron concentration of each solution was 0.052 M. Considering that TCM containing borax is mostly taken by patients at room temperature, an ^11^B NMR study was conducted in an environment of 278 K–323 K. Unlike the ^11^B NMR study of borax solutions at varying pHs, according to Figure 3, it can be seen that the effect of temperature on the ^11^B NMR spectrum of borax solutions is not significant and can be ignored.

### 2.2. Borax Conversion to Boric Acid Studies Vinegar Processing of Borax Studies

The preparation method of borax has been widely recorded in medical prescriptions and TCM medicine throughout history. The prescription for using borax as medicine has been recorded as early as the Song Dynasty, and the Taiping Shenghui Formula alone includes “finely grind” and “vinegar to remove stones, boil into frost” [40]. We have studied the preparation of borax by the vinegar processing method, which is a common method for preparing borax of TCM, and observed the changes in the form of boron elements through ^11^B NMR experiments.

The ^11^B NMR experiment was conducted on a mixed sample of borax and 5% acetic acid, and it was found that borax can react with acetic acid at room temperature to generate boric acid. Referring to the reaction between borax and hydrochloric acid, it is speculated that the reaction equation and ion equation involved are as follows:

Na_2_B_4_O_7_ + 2CH_3_COOH + 5H_2_O→4H_3_BO_3_ + 2CH_3_COONa (1)

B_4_O_7_^2−^ + 2H^+^ + 5H_2_O→4H_3_BO_3_ (2)

**Figure 4:**
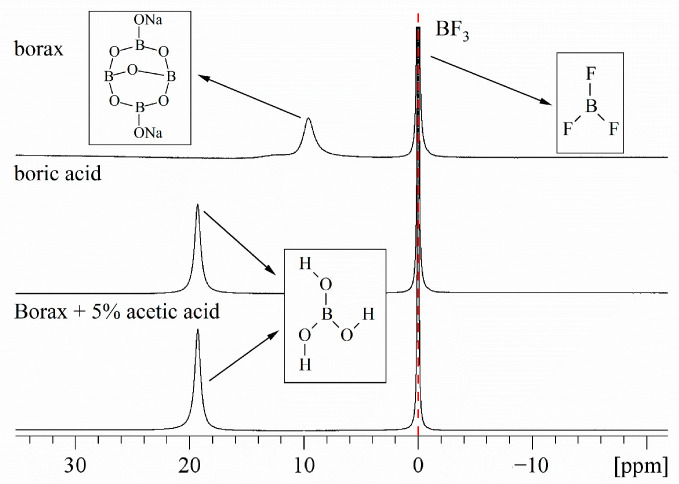
^11^B NMR spectra of borax, boric acid, and borax + 5% acetic acid samples, The reference signal of BF_3_ at δ = 0.00 ppm is shown.

Figure 4 shows ^11^B NMR spectra of borax, boric acid, and borax + 5% acetic acid samples. The reaction is rapid and the generation of boric acid is stable. Borax and acetic acid stably generate boric acid in an aqueous solution, and the boric acid peak is detected by ^11^B NMR. After 8h, the boric acid peak is still determined.

### 2.3. ^11^B NMR of Boron-Containing TCM

The initial screening of 20 commercially available TCMs containing boric acid or borax was performed using the ^11^B NMR technique without external standards, and the results are shown in Table 1.

The preliminary screening experiment found that not all of the 20 boron-containing TCMs preparations sold on the market still contained boron after processing, which means that the active ingredient in them may not be related to boron. The analysis of 14 detected boron signal drugs is as follows: under the existing detection conditions, only one signal peak was detected in the ^11^B NMR spectrum of QingYanWan, Boric Acid Ear Drops, Lotin Boracic Acid, and QingYanPian. Combined with the Chemical shift of boric acid, this should be a boric acid signal peak. Therefore, boric acid is contained in all four traditional Chinese medicine preparations. It is worth noting that boric acid signals were detected in QingYanWan and QingYanPian with only borax added. It is speculated that the possible source of boric acid is borax’s generation of boric acid during the traditional Chinese medicine processing and in the formula, which needs further verification. Only the presence of borax was detected in ZhenZhuBingPengSan and BingPengSan. The presence of borax and a new water-soluble boron-containing compound were not only detected in DingPeng Cream, Laryngitis Pill, and ZhuHuangChuiHouSan; and not only was the presence of borax detected in the Compound Borax Solution, but two new water-soluble boron compounds were also detected. No presence of borax was detected in BingPengYanHouSan and BeiLing Capsules, but a new water-soluble boron-containing compounds were detected; Three new water-soluble boron-containing compounds were detected in the BingPengHanPian; Boric Acid Ointment did not detect the presence of boric acid, but two chloroform soluble boron-containing compounds were detected. The summary of ^11^B NMR signals detected in 14 boron-containing traditional Chinese medicine preparations is shown in Table 2.

## 3. Discussion

**Figure 5:**
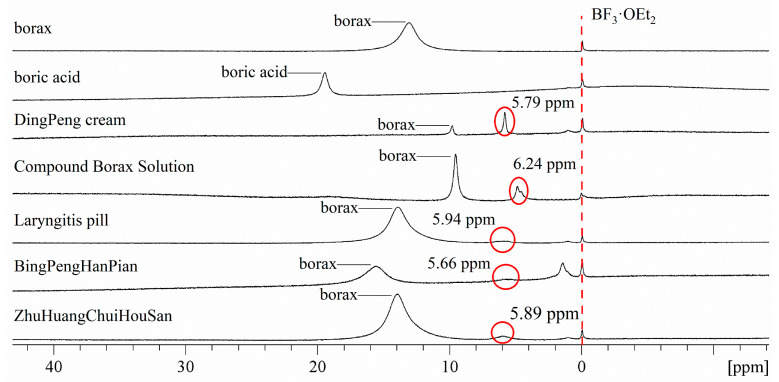
^11^B NMR-superimposed spectra of DingPeng cream, Compound Borax Solution, Laryngitis Pill, BingPengHanPian, and ZhuHuangChuiHouSan.

We discuss five of these TCMs preparations, from No.7 to 11, in Table 2, and their ^11^B NMR-superimposed spectra with boric acid and borax are shown in Figure 5. The query in the TCM prescription database revealed that the main boron-containing TCM added to these five boron-containing TCMs was borax, and in the NMR-superimposed spectra of ^11^B in Figure 5, the raw material borax peak was detected in all the other four TCMs except the Ice boron tablets and the similar peak in Ice boron tablets was shifted to δ_B_ = 15.4 ppm in the lower field, which was beyond the range of δ_B_ 9.80–13.09 ppm defined for borax in the previous part of our experiments. This may be the effect of the concentration or pH [33].

In addition, we focus more on the discussion of the signal peak δ_B_ 5.6–6.2 ppm in these five boron-containing TCMs. PH testing was conducted on these five drugs, and the pH of DingPeng cream was measured to be 9.2, Compound Borax Solution was measured to be 9.5, Laryngitis Pill and BingPengHanPian were measured to be 9.0, and ZhuHuangChuiHouSan was measured to be 8.0. The relationship between the pH and chemical shift of the above TCM is roughly consistent with the hypothesis of the ^11^B NMR study of borax solutions at various pHs (2.1.2) in this article. However, the borax raw material peaks of these five TCMs still exist, which is inconsistent with the experimental results in Section 2.1.2. Pople et al. [41] showed that if the mean lifetime of an atom remaining on one of the two different sites is long compared with the inverse of the chemical shift difference between the two sites, the spectrum will consist of two lines. On the other hand, if the characteristic exchange time is short compared with the difference, a single line with a weighted mean frequency will be observed. This may be the reason why these five TCMs are still able to detect the borax peak. TCM-containing boron has complex ingredients and various processing methods. There may be some factors that affect the coexistence of SP^2^ borate and SP^3^ metaborate in alkaline solutions, which can be detected simultaneously by ^11^B NMR.

On the other hand, boron-containing traditional Chinese medicine has complex components, including traditional Chinese medicine herbs with lipid or halogen components. We speculate that it also may be the C_3_B←NR_3_, Hal_3_←NR_3_, Hal_3_B←OR_2_, (Car-)Boranes, BHal_3_ structure according to the reported information [7,14,39,42,43], considering the obvious signals that DingPeng Cream and Compound Borax Solution are in a liquid phase in nature. Both contain certain glycerol and carbonate; we prefer the structure of Hal_3_B←OR_2_, which remains to be verified by our further experiments.

These boron-containing compounds, which are not borax or boric acid, maybe the reason for the different efficacy of each formulation and also provide a new idea for the improvement of our TCM formulations, that is, if the morphological changes of borax or boric acid in the formulations are obtained and the structures of the newly produced boron-containing compounds are analyzed, it is possible to avoid adding boric acid and borax and add these boron-containing compounds directly, which has great significance for the safety of TCM formulations.

## 4. Materials and Methods

### 4.1. Materials

D_2_O was purchased from the Qingdao Longteng Company (Qingdao, China), DMSO and CDCl_3_ from the CIL Company (Cambridge, CA, USA), and BF_3_ from the Aladdin Company (Shanghai, China). Twenty kinds of boron-containing TCM were all commercially available.

### 4.2. Methods

#### 4.2.1. ^11^B NMR Analysis

The NMR spectrometer was a Bruker ADVANCE III HD 400 MHz, the probe was a PA BBO 400S1 BBF-H-D-05 Z SP probe, the ^11^B resonance frequency was 128.38 MHz, the experimental temperature was 298 K, the ^11^B NMR experimental spectral width (SW) was 198 ppm, the sampling time was 1.28 s, and the number of scans NS was 128. The zg pulse sequence was used and the NMR spectra processing was carried out by Bruker Topspin 4.1.1. NMR spectroscopy was manually corrected for baseline and phase, and calibrated to BF_3_·OEt_2_ at δ0.00 ppm for D_2_O.

#### 4.2.2. NMR Sample Preparation of Boron-Containing TCMs

The 20 boron-containing TCMs were as follows: MaYingLongBaBaoYanGao, LuPaoSan, ShuJinDingTong Capsules, HouKangSan, BoYunDing, ZhenZhuBingPengSan, BingPengHanPian, QingYanWan, ZhuHuangChuiHouSan, Laryngitis Pill, BeiLing Capsules, BingPengSan, DingPeng Cream, QingYanPian, and BingPengSan. Twenty TCMs were dissolved in D_2_O (0.6 mL) with maximum solubility, sonicated for 1 min, vortexed and mixed, centrifuged, and the supernatant was 0.5 mL transferred to a 5 mm NMR sample tube.

Boric Acid Ear Drops (60 mg), Lotin Boracic Acid (2 g), Naphazoline Hydrochloride (1 g), and Compound Borax Solution (200 mg) were lyophilized and concentrated and dissolved in DMSO (0.6 mL), sonicated for 1 min, vortexed and mixed, centrifuged, and 0.5 mL of supernatant was transferred to 5 mm NMR sample tubes.

Boric Acid Ointment (50 mg) was dissolved in CDCl_3_ (0.6 mL) and sonicated for 1 min, vortexed and mixed, centrifuged, and 0.5 mL of supernatant was transferred to a 5 mm NMR sample tube.

#### 4.2.3. NMR Sample Preparation of Boric Acid and Borax

For the boric acid standard solution preparation, 1 mg, 1.5 mg, 2.5 mg, 3.5 mg, 5 mg, 7.5 mg, and 10 mg of weighed boric acid were dissolved in 0.5 mL of D_2_O.

For the borax standard solution preparation, 1.5 mg, 2.5 mg, 3.5 mg, 5 mg, 7.5 mg, and 10 mg of weighed borax were dissolved in 0.5 mL of D_2_O. All relevant standard solutions in the experiments of this paper were prepared without using pH buffer solutions.

^11^B NMR solutions of borax were prepared at various pHs: 2, 3, 4, 5, 6, 7, 8, 9, 10, 11, and 12. A weighed amount of borax was dissolved in a D_2_O solution, After borax had been completely dissolved, the pH of the solution was adjusted with NaOH or acetic acid. The pH detection of borax aqueous solution was carried out using pH precision test strips. For the ^11^B NMR study of borax solutions at various temperatures, 10 mg of borax was dissolved in 0.5 mL of D_2_O. The temperature control switch of the NMR spectrometer was turned on and an ^11^B NMR experiment was conducted after stabilizing at the target temperature.

## 5. Conclusions

Since boron-based compounds have been revealed to be non-toxic nowadays, boron-based drugs have also been developed and approved one after another [44,45,46], and boron-containing TCM, as a major category of TCM, is also worth paying attention to. First, the ^11^B NMR technology in this study was verified as a potential method for the first time for detecting boron in various commonly sold boron-containing TCM formulations. Secondly, this study found that the ^11^B NMR chemical shift of TCM borax aqueous solution is influenced by concentration, i.e., pH, with little effect on temperature. The reason for the change in ^11^B NMR chemical shift is related to the elemental form of boron in borax aqueous solution. The increase in the number of SP^3^ hybridized boron atoms after coordination will cause the ^11^B NMR chemical shift to shift towards higher fields. Then, the common processing method for TCM borax, vinegar preparation, can convert borax into boric acid. Finally, this study conducted a preliminary study on the elemental forms of boron in various common boron-containing TCM compounds based on ^11^B NMR technology and found that some boron-containing TCM has the same signal peaks of δ_B_ 5.6–6.2 ppm. This indicates that it may be a new and effective non-borax or boric acid coordinated boron-containing compound, which may also be a boron-containing compound influenced by other lipids and halogens in relevant prescriptions.

^11^B NMR has the significant advantages of simple operation and no sample damage, and it will be important to determine boron-based compounds of TCM, which could open a new avenue of research methods for boron-containing TCM fingerprints and the identification of effective boron-containing components. This article is the first to apply ^11^B NMR to the study of boron-containing TCM and proposes the possibility of the evolution of effective boron containing active small molecules. More research is needed to understand the specific chemical structure of boron-containing compounds, to serve as the basis for the development of new boron-containing drugs.

## Figures and Tables

**Table 1:** Summary of the number of peaks in ^11^B NMR spectra of 20 commercially available TCM.

No.	Proprietary Chinese Medicine Preparation	The Number of ^11^B Signal Peaks
1	Compound Borax Solution	3
2	BingPengHanPian	3
3	ZhuHuangChuiHouSan	3
4	Laryngitis Pill	3
5	DingPeng Cream	3
6	Boric Acid Ointment	2
7	ZhenZhuBingPengSan	1
8	QingYanWan	1
9	Boric Acid Ear Drops	1
10	Lotion Boracic Acid	1
11	BeiLing Capsules	1
12	BingPengYanHouSan	1
13	QingYanPian	1
14	BingPengSan	1
15	MaYingLongBaBaoYanGao	—
16	LuPaoSan	—
17	ShuJinDingTong Capsules	—
18	HouKangSan	—
19	BoYunDing	—
20	Naphazoline Hydrochloride	—

**Table 2:** Summary of the position of peaks in ^11^B NMR spectra of 20 commercially available TCM.

No.	Proprietary Chinese Medicine Preparation	Borax Peak	Boric Acid Peak	δ_B_ 1–2 ppm	δ_B_ 5.6–6.2 ppm	δ_B_ = 15.4 ppm	δ_B_ 18–19 ppm	δ_B_ = 23.3 ppm
1	QingYanWan		√					
2	Boric Acid Ear Drops		√					
3	Lotion Boracic Acid		√					
4	QingYanPian		√					
5	ZhenZhuBingPengSan	√						
6	BingPengSan;	√						
7	DingPeng Cream	√		√	√			
8	Compound Borax Solution	√			√		√	
9	Laryngitis Pill	√		√	√			
10	BingPengHanPian			√	√	√		
11	ZhuHuangChuiHouSan	√		√	√			
12	BingPengYanHouSan			√				
13	BeiLing Capsules						√	
14	Boric Acid Ointment						√	√

The chemical shift range of borax in the ^11^B NMR spectrum is tentatively set at 9.80~13.09 ppm; the chemical shift of boric acid is about 19.44 ppm.

## Data Availability

Data are contained within the article.
